# Start at the Center: Age-Friendly Public Health Systems and Healthy Brain Initiative Frameworks

**DOI:** 10.1093/geroni/igaa057.2545

**Published:** 2020-12-16

**Authors:** Megan Wolfe, Molly French, John Shean

**Affiliations:** 1 Trust for America's Health, Washington, District of Columbia, United States; 2 Alzheimer's Association, Washington, District of Columbia, United States

## Abstract

SIGNIFICANCE. Older adults can contribute wisdom, skills, and time to communities. The public health sector has unique capabilities for creating conditions that promote health, foster community connections, and quality of life. METHODS. Two frameworks provide public health (PH) with core strategies to improve outcomes for all older adults. The Framework for Creating an Age-Friendly Public Health System (AFPHS) supports the PH role, as demonstrated by 37 of Florida’s 67 county health departments that are piloting the AFPHS Framework. The Healthy Brain Initiative’s (HBI) State and Local Public Health Partnerships to Address Dementia is a framework for action used by PH to promote cognitive health, improve care for cognitive impairment, and increase caregiving supports. Both frameworks call for utilizing regional data and cross-sector partnerships. IMPLICATIONS. PH can contribute to community-wide initiatives to promote well-being and community connections for older adults. Cross-sector partnerships can start by using available tools and planning guides.

